# The Marine-Derived Triterpenoid Frondoside A Inhibits Thrombus Formation

**DOI:** 10.3390/md18020111

**Published:** 2020-02-14

**Authors:** Emmanuel Ampofo, Thomas Später, Lisa Nalbach, Michael D. Menger, Matthias W. Laschke

**Affiliations:** Institute for Clinical & Experimental Surgery, Saarland University, 66421 Homburg/Saar, Germany; thomas.spaeter@uks.eu (T.S.); lisa.nalbach@uks.eu (L.N.); michael.menger@uks.eu (M.D.M.); matthias.laschke@uks.eu (M.W.L.)

**Keywords:** frondoside A, thrombus formation, thrombosis, hemostasis, platelets, PI3K, dorsal skinfold chamber

## Abstract

Background: The marine-derived triterpenoid frondoside A inhibits the phosphatidylinositol-3-kinase (PI3K) pathway in cancer cells. Because this pathway is also crucially involved in platelet activation, we studied the effect of frondoside A on thrombus formation. Methods: Frondoside A effects on platelet viability, surface adhesion molecule expression, and intracellular signaling were analyzed by flow cytometry and Western blot. The effect of frondoside A was analyzed by photochemically induced thrombus formation in the mouse dorsal skinfold chamber model and by tail vein bleeding. Results: Concentrations of up to 15 µM frondoside A did not affect the viability of platelets, but reduced their surface expression of P-selectin (CD62P) and the activation of glycoprotein (GP)IIb/IIIa after agonist stimulation. Additional mechanistic analyses revealed that this was mediated by downregulation of PI3K-dependent Akt and extracellular-stimuli-responsive kinase (ERK) phosphorylation. Frondoside A significantly prolonged the complete vessel occlusion time in the mouse dorsal skinfold chamber model of photochemically induced thrombus formation and also the tail vein bleeding time when compared to vehicle-treated controls. Conclusion: Our findings demonstrated that frondoside A inhibits agonist-induced CD62P expression and activation of GPIIb/IIIa. Moreover, frondoside A suppresses thrombus formation. Therefore, this marine-derived triterpenoid may serve as a lead compound for the development of novel antithrombotic drugs.

## 1. Introduction

Thrombus formation is triggered by intravascular blood clotting as a result of hyperaggregability of platelets, increased blood viscosity, and impaired fibrinolysis. Antithrombotic compounds can be divided into two classes, anticoagulants and antiplatelet drugs [1]. Antiplatelet drugs target specific surface proteins, including adenosine diphosphate (ADP) and glycoprotein (GP)IIb/IIIa receptors, as well as intracellular proteins, such as cyclooxygenase [2]. These drugs bear an increased risk of bleeding and antiplatelet drug resistance. Therefore, the identification of novel compounds with improved efficacy and safety is of major interest for the treatment of thrombotic diseases.

Platelet activation is induced by various phospho-regulated pathways, including phosphatidylinositol-3-kinase (PI3K) [3]. All PI3K isoforms are expressed in platelets, and agonist-induced platelet stimulation leads to the generation of phosphatidylinositol 3,4,5-trisphosphate (PIP3) [4]. This, in turn, results in Akt and extracellular-stimuli-responsive kinase (ERK)1/2 phosphorylation as well as subsequent platelet activation [4,5,6]. Activated platelets express specific proteins, such as GPIIb/IIIa and P-selectin on their surface, which mediate platelet aggregation and hemostatic plug formation [7,8,9,10].

**Figure 1:**
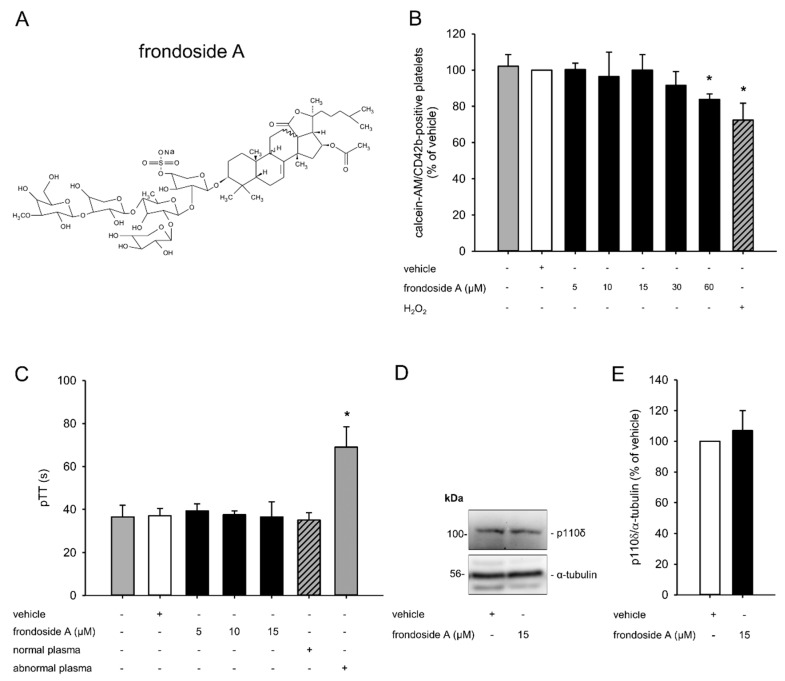
Effect of frondoside A on platelet viability. (**A**) Molecule structure of frondoside A. (**B**) Platelet-rich plasma (PRP) was incubated with different concentrations of frondoside A (black bars, *n* = 5) or vehicle (white bar, *n* = 5) for 30 min. Untreated PRP served as negative control (grey bar, *n* = 5). H_2_O_2_-treated PRP served as positive control (shaded gray bar, *n* = 5). Platelet viability was assessed by flow cytometry. Data are given in % of vehicle. Mean ± SD. * *p* < 0.05 vs. vehicle. (**C**) Platelet-poor plasma (PPP) was incubated with different concentrations of frondoside A (black bars, *n* = 5) or vehicle (white bar, *n* = 5) for 30 min and pTT was assessed by the determination of clotting time. Untreated PPP (gray bar, *n* = 5) and normal plasma (shaded gray bar, *n* = 5) served as negative controls. Abnormal plasma (dotted gray bar, *n* = 5) served as positive control. Mean ± SD. * *p* < 0.05 vs. vehicle. (**D**) PRP was incubated with frondoside A (15 µM) or vehicle for 30 min, and the expression of the PI3K subunit p110δ and α-tubulin was analyzed by Western blot. (**E**) Quantitative analysis of the PI3K subunit p110δ expression (black bar, frondoside A; white bar, vehicle; *n* = 3). Data are given in % of vehicle. Mean ± SD.

Frondoside A, a triterpenoid, was originally isolated from an extract of the Atlantic sea cucumber *Cucumaria frondosa*, and cannot chemically be synthesized due to its complex structure [11,12] (Figure 1A). Several studies have reported that frondoside A exerts an antiproliferative and pro-apoptotic activity [13,14,15,16]. Moreover, this compound is capable of potentiating the anticancer effects of conventional cytotoxic drugs such as gemcitabine and 5-fluorouracil [13,16]. Detailed molecular analyses revealed that these effects are mainly mediated by the inhibition of PI3K, resulting in reduced Akt signaling and caspase-induced cell death [17,18,19]. 

Based on the inhibitory properties of frondoside A on PI3K signaling, we assumed that this compound exerts an antithrombotic activity. To test this hypothesis, we first investigated the effects of frondoside A on platelet viability, activated partial thromboplastin time (pTT), and platelet activation. Furthermore, we analyzed the antithrombotic effect of frondoside A in vivo on photochemically induced thrombus formation in the mouse dorsal skinfold chamber model and on prolongation of tail vein bleeding time. 

## 2. Results

### 2.1. Effect of Frondoside A on Platelet Viability 

In a first set of experiments, we determined suitable concentrations of frondoside A to exclude the possibility that the analyzed effects of this compound may be due to platelet toxicity. For this purpose, platelet-rich plasma (PRP) was incubated with different concentrations of frondoside A ranging from 5 µM to 60 µM and the viability of calcein-acetomethoxy (AM)/CD42b-stained platelets was analyzed by means of flow cytometry. These analyses demonstrated that concentrations up to 15 µM did not exert any toxic effect on platelets (Figure 1B). In contrast, 30 µM slightly and 60 µM significantly reduced platelet viability. Accordingly, we used concentrations of 5–15 µM frondoside A for the following experiments.

Next, we investigated whether non-toxic concentrations of frondoside A affected the intrinsic coagulation cascade by assessing the pTT. Normal and abnormal plasma served as positive and negative controls, respectively, in this experimental setting. We found that the compound did not prolong the pTT when compared to vehicle (Figure 1C). 

PI3K is a major regulator of platelet activation. To exclude the possibility that frondoside A affects the expression of this kinase, we performed additional Western blot analyses. These analyses demonstrated that a concentration of 15 µM frondoside A did not attenuate the expression of PI3K, as shown by a comparable level of the PI3K subunit p110δ in frondoside-A- and vehicle-treated PRP (Figure 1D,E). 

### 2.2. Effect of Frondoside A on Platelet Activation 

**Figure 2:**
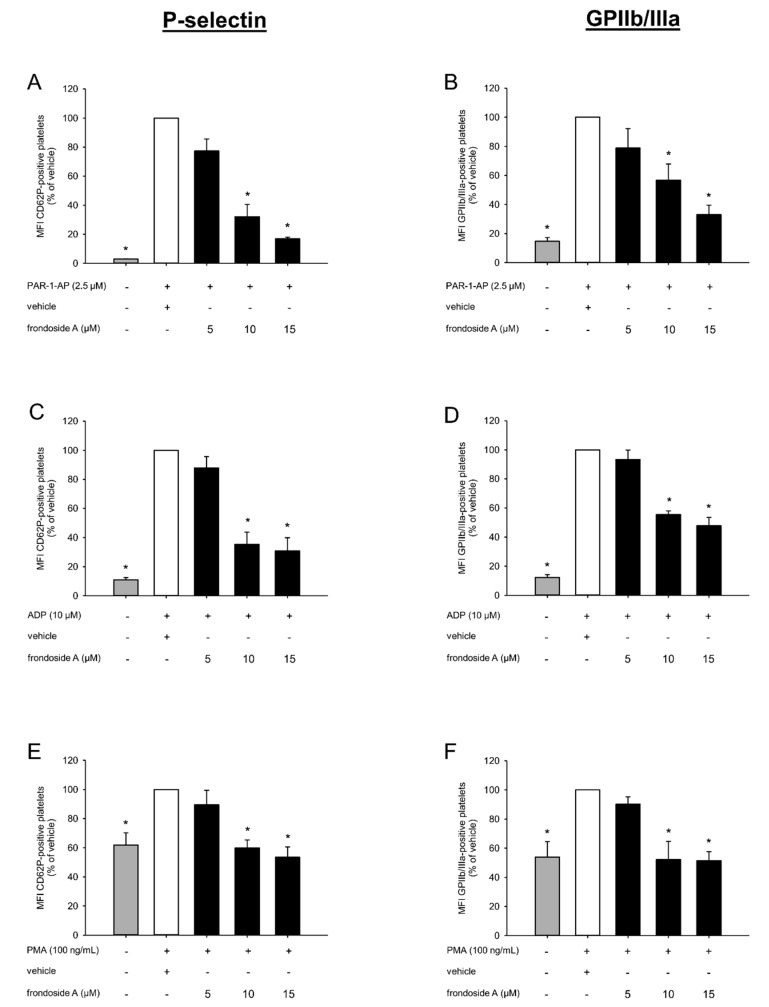
Effect of frondoside A on platelet activation. (**A**–**F**) PRP was incubated with different concentrations of frondoside A (black bars, *n* = 5) or vehicle (white bars, *n* = 5) for 30 min following stimulation with PAR-1-AP (**A**,**B**), ADP (**C**,**D**), or PMA (**E**,**F**) for 10 min. Surface levels of activated GPIIb/IIIa and CD62P were assessed by means of flow cytometry. Unstimulated PRP served as negative control (gray bars, *n* = 5). Data are given in % of vehicle. Mean ± SD. * *p* < 0.05 vs. vehicle.

Platelet aggregation is mediated by specific surface adhesion molecules. To analyze the effect of frondoside A on this process, the expression and activation of the two adhesion proteins P-selectin (CD62P) and GPIIb/IIIa were analyzed by flow cytometry. For this purpose, PRP was incubated with different concentrations of frondoside A for 30 min, followed by agonist stimulation with protease-activated receptor-1-activating peptide (PAR-1-AP), ADP, and phorbol-12-myristate-13-acetate (PMA). As expected, the three agonists markedly increased the surface expression of P-selectin (Figure 2A,C,E) and the activity of GPIIb/IIIa (Figure 2B,D,F). It was of note that frondoside A concentration-dependently decreased the expression of P-selectin and suppressed the conformal switch of inactive GPIIb/IIIa into its active form (Figure 2A–F). 

### 2.3. Effect of Frondoside A on PI3K Signaling 

**Figure 3:**
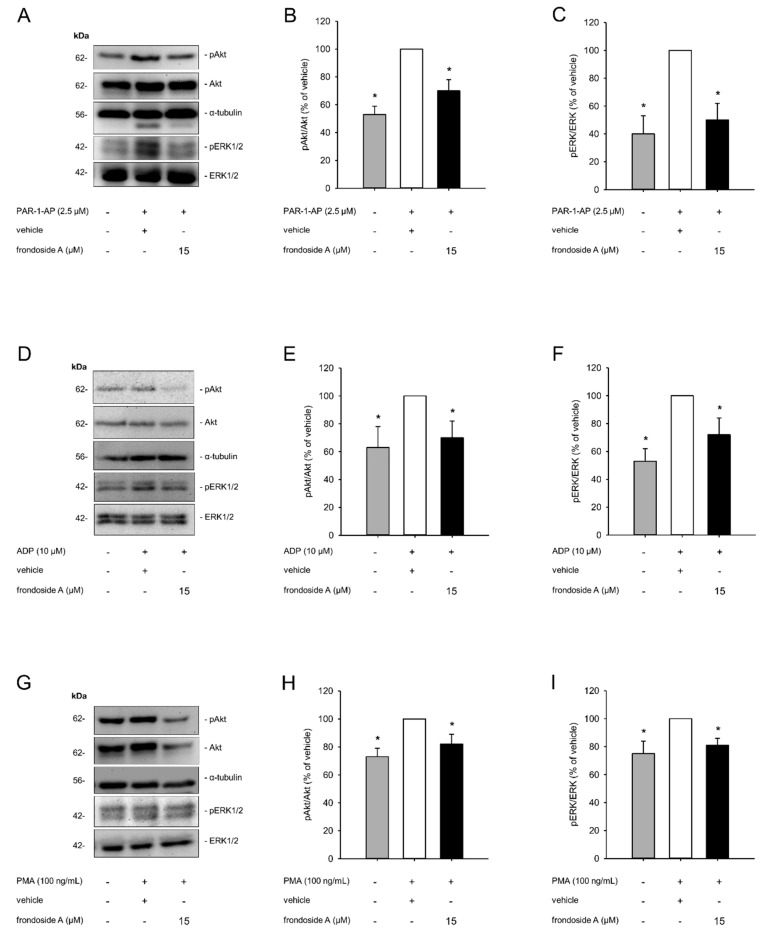
Effect of frondoside A on PI3K signaling. (**A**–**I**) PRP was incubated with frondoside A (15 µM) or vehicle for 30 min followed by stimulation with PAR-1-AP (**A**), ADP (**D**), or PMA (**G**) for 10 min. Untreated PRP served as negative control. The expressions of pAkt, Akt, pERK1/2, ERK1/2, and α-tubulin were analyzed by means of Western blot. Quantitative analysis of pAkt/Akt expression (**B**,**E**,**H**) and pERK/ERK expression (**C**,**F**,**I**) (black bars, frondoside A; white bars, vehicle; gray bars, untreated control; *n* = 3) of (**A**), (**D**) and (**G**), respectively. Data are given in % of vehicle. Mean ± SD. * *p* < 0.05 vs. vehicle.

Previous studies have demonstrated that frondoside A is capable of inhibiting the PI3K pathway, which is known to be crucial for platelet activation. Therefore, we further investigated the influence of this compound on the activity of the PI3K downstream effectors Akt and ERK1/2. Western blot analyses revealed that agonist-induced platelet activation increased the phosphorylation of Akt and ERK1/2, which, in turn, was suppressed by frondoside A (Figure 3A–I). These findings clearly indicated that frondoside A inhibits platelet activation by interfering with the PI3K pathway.

### 2.4. Effect of Frondoside A on Thrombus Formation In Vivo 

**Figure 4:**
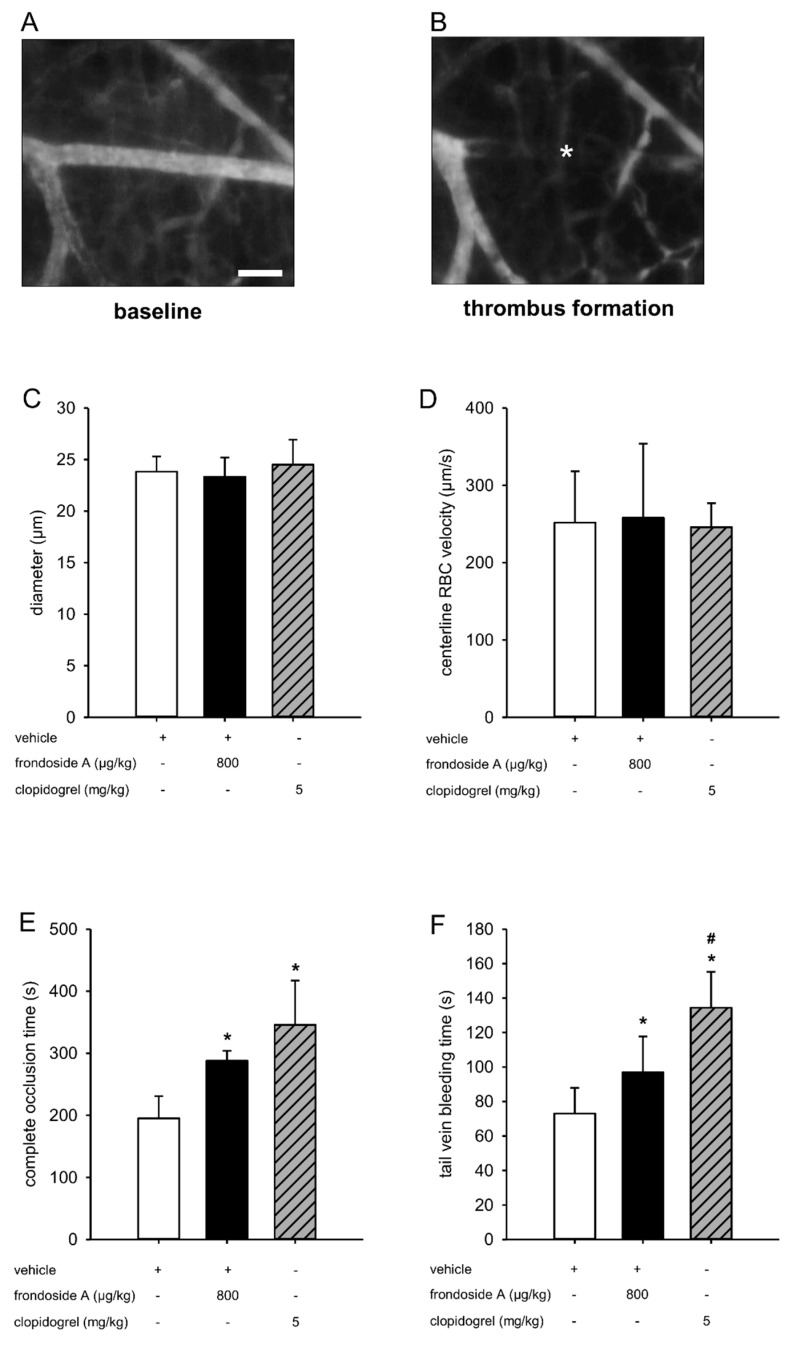
Effect of frondoside A on thrombus formation in vivo. (**A**,**B**) Intravital fluorescent microscopic images of a postcapillary venule within the dorsal skinfold chamber of a vehicle-treated mouse before (baseline) and after photochemically induced thrombus formation (asterisk). Blue-light epi-illumination with contrast enhancement by 5% FITC-labeled dextran 150,000 Da. Scale bar: 50 µm. (**C**,**D**) Diameter (**C**) and centerline RBC velocity (**D**) of postcapillary and collecting venules within the dorsal skinfold chamber of mice treated with frondoside A (black bars, *n* = 6), vehicle (white bars, *n* = 6) and clopidogrel (shaded gray bar, *n* = 6). Mean ± SD. (**E**) Complete occlusion time of postcapillary and collecting venules upon photochemically induced thrombus formation in dorsal skinfold chambers of mice treated with frondoside A (black bar, *n* = 6), vehicle (white bar, *n* = 6) and clopidogrel (shaded gray bar, *n* = 6), as assessed by intravital fluorescence microscopy. Mean ± SD. * *p* < 0.05 vs. vehicle. (**D**) Tail vein bleeding time of mice treated with frondoside A (black bar, *n* = 6), vehicle (white bar, *n* = 6) and clopidogrel (shaded gray bar, *n* = 6). Mean ± SD. * *p* < 0.05 vs. vehicle. ^#^ *p* < 0.05 vs. frondoside A.

Finally, we tested the antithrombotic activity of frondoside A on photochemically induced thrombus formation in the dorsal skinfold chamber model (Figure 4A,B). For this purpose, BALB/c mice were treated with frondoside A and vehicle as well as clopidogrel, which was chosen as a positive control due to its well-known antithrombotic activity [20]. Subsequently, the complete vessel occlusion time of postcapillary and collecting venules was measured. To exclude the possibility that this measurement was affected by altered microcirculatory conditions, we first analyzed the diameters and centerline red blood cell (RBC) velocities of these vessels. This analysis did not show any differences between the three groups (Figure 4C,D). In line with our in vitro results, we further found that frondoside A significantly prolonged the vessel occlusion time (Figure 4E). Moreover, frondoside A also significantly prolonged the tail vein bleeding time when compared to vehicle-treated controls (Figure 4F). 

## 3. Discussion

Marine-derived compounds provide many of the lead structures used as templates for the generation of novel molecules with enhanced biological activity. Several of these compounds are already being used for the treatment of different pathological conditions, including cancer, viral and fungal infections, inflammatory diseases, and thrombosis [21]. The latter is mainly controlled by phospho-regulated signaling transductions, such as the PI3K pathway. In this study, we identified the triterpenoid frondoside A from the Atlantic sea cucumber *Cucumaria frondosa* as a novel antithrombotic compound which is capable of reducing agonist-induced platelet activation via the inhibition of this pathway. 

We first determined suitable non-toxic concentrations of frondoside A. We found that concentrations of 5–15 µM frondoside A did not affect the viability of platelets. In contrast, Dyshlovoy et al. demonstrated that treatment of the human prostate cancer cell lines DU145 and PC3 with much lower concentrations of only 1–2 µM frondoside A already induced caspase3/7 and PARP cleavage, resulting in a reduced cell viability [14]. These contradictory findings may be explained by a different sensibility of the cell types used. Moreover, the binding to soluble proteins within PRP, such as human serum albumin (HSA) or immunoglobulins, may have interfered with the uptake of frondoside A into platelets [22]. Therefore, higher concentrations of the compound were needed to exert detectable antithrombotic effects in our experimental setting. 

Primary hemostasis is triggered by various signaling pathways within platelets. Several studies have shown that the class I PI3Ks (PI3Kα, PI3Kβ, PI3Kγ, and PI3Kδ) are the most important mediators in these cells, leading to platelet activation, spreading, and aggregation [3]. Although we found that frondoside A does not affect the expression of PI3K, it markedly reduced the activity of the PI3K pathway, as shown by a diminished agonist-induced phosphorylation of Akt and ERK1/2. This was in line with the results of Park et al. showing that frondoside A significantly inhibited PI3K/Akt, ERK1/2, and p38 MAPK activation in PMA-stimulated breast cancer cells [18]. 

Under hemostatic conditions, platelets do not bind to other cell types or to soluble plasma proteins. However, when activated, they strongly interact with leukocytes, von Willebrand factor (vWF), and fibrinogen via specific surface adhesion molecules [23]. Accordingly, we investigated the inhibitory effect of frondoside A on the expression of two major platelet adhesion molecules. Flow cytometric analyses revealed that 5–15 µM of the compound reduced the agonist-induced expression of P-selectin and prevented the shift of GPIIb/IIIa from a low- to a high-affinity state following PAR-1-AP stimulation. This concentration-dependent reduction was not observed between a frondoside A concentration of 10 and 15 µM following ADP and PMA stimulation. These contradictory findings may be explained by the fact that PAR-1-AP only activates the Gq-coupled receptor [24]. Gq, in turn, activates phospholipase (PLC)-β, leading to platelet shape change, granule secretion, and amplification of platelet activation [25]. In contrast, ADP activates platelets through both the Gq- and Gi-coupled receptor, whereas PMA solely activates the Gi-coupled receptor [26,27]. The latter induces the activation of GPIIb/IIIa and stabilizes platelet aggregation [25]. Our results indicated that 10 µM frondoside A seems to be sufficient to completely suppress Gi signal transduction, while a higher concentration of 15 µM is necessary to efficiently inhibit the Gq-coupled receptor. It should be considered that frondoside A may also affect the expression of other adhesion molecules, such as GPIb/V/IX. This receptor mediates the adhesion of platelets to exposed extracellular matrix (ECM) compounds from the disrupted endothelial lining [28]. Other interesting candidates may be the integrins α_2_β_1_ and α_V_β_3_, which induce platelet activation and aggregation by interaction with collagen and RGD-containing proteins [28]. 

For our in vitro experiments, we used the agonists PAR-1-AP and ADP for PI3K-mediated platelet activation. In addition, platelets were stimulated with PMA, which is a strong protein kinase (PK)C agonist [29]. It was of note that frondoside A decreased platelet activation induced by all three agonists. Hence, our findings indicate that PKC may also be an important target of frondoside A. Besides a direct inhibition of this kinase, frondoside A may alternatively affect signaling pathways involved in the regulation of PKC expression and activation. 

Finally, we assessed the antithrombotic effect of frondoside A in a well-established in vivo model. For this purpose, thrombus formation was induced in postcapillary and collecting venules within mouse dorsal skinfold chambers by administration of the fluorescent dye fluorescein isothiocyanate (FITC)-labeled dextran 150,000 and blue light exposure. This leads to the disruption of the endothelial lining by local generation of reactive oxygen species [30,31]. As expected, we measured a prolonged complete vessel occlusion time in animals treated with frondoside A when compared to vehicle-treated controls. Moreover, similar to the positive control used herein, clopidogrel [20,32,33,34], the compound increased the tail vein bleeding time.

The PI3K pathway has been shown to be a dominant signaling pathway in different nucleate cell types, which regulates the balance between apoptosis and survival [34]. Several groups have reported that frondoside A exerts potent anti-cancer effects in vitro and in vivo [19,35]. It is conceivable that this compound also affects the viability of non-malignant cells, such as platelets. However, Marzouqi et al. [36] showed that the treatment of mice with frondoside A over 24 days did not influence the number of white blood cells, red blood cells, or platelets. Based on this finding, we assumed that the prolonged blood vessel occlusion time observed herein was caused by a suppressed platelet activity rather than a reduced platelet viability.

The suppression of platelet activation by frondoside A may be mediated by different mechanisms. In addition to its well-studied inhibitory effect on the ATP-stimulated phosphorylation of several kinase pathways, including PI3K [17], frondoside A has been shown to block the activity of the multidrug-resistant P-glycoprotein by direct interaction [37]. Platelets also express different glycoproteins on their surface. Hence, frondoside A may suppress platelet function by binding to these surface proteins. On the other hand, the steroidal backbone of the molecule may also affect platelet activity. In line with this assumption, it has been reported that estrogen decreases inositol 1,4,5-triphosphate (IP3) and increases cAMP levels in ADP-treated platelets, resulting in reduced platelet activation [38]. Moreover, progesterone and estradiol suppress agonist-induced platelet aggregation [39]. The modification of specific chemical groups of frondoside A could improve the antithrombotic activity. However, it is not possible to synthesize this molecule due to its complex structure. Therefore, further studies are required not only to clarify the exact mode of action of frondoside A, but also to develop synthesis methods for frondoside A.

In summary, our findings demonstrated that frondoside A is a potent inhibitor of the PI3K pathway in platelets and, thus, suppresses thrombus formation. Hence, this marine-derived triterpenoid may serve as a lead compound for the development of novel antithrombotic drugs. 

## 4. Materials and Methods

### 4.1. Chemicals, Reagents, and Antibodies

Frondoside A, ADP, PAR-1-AP, PMA, FITC-labeled dextran 150,000, and rhodamine 6G were purchased from Sigma-Aldrich (Taufkirchen, Germany), calcein-AM from Molecular Probes (Eugene, OR, USA), clopidogrel from Sanofi (Frankfurt, Germany), ketamine (Ursotamin^®^) from Serumwerke Bernburg (Bernburg, Germany), and xylazine (Rompun^®^) from Bayer (Leverkusen, Germany). Anti-CD42b, anti-CD62P, anti-GPIIb/IIIa, and IgG1-κ isotype controls were purchased from BD Biosciences (Heidelberg, Germany). The antibodies against α-tubulin (sc-53646) and phospho (p)-Akt1/2/3 (sc-33437) were obtained from Santa Cruz Inc. (Heidelberg, Germany). The antibodies against p-ERK (50011) and anti-ERK (115799) were purchased from Abcam (Cambridge, UK). The antibody against anti-Akt1/2/3 (11E7) was purchased from Cell Signaling (Frankfurt am Main, Germany).

### 4.2. Ethics Statement

For the isolation of PRP, venous blood was drawn from six healthy volunteers after obtaining their written informed consent and with the approval of the local ethics review board.

### 4.3. Preparation of PRP and Platelet Poor Plasma (PPP)

In order to prepare PRP, venous blood was drawn into plastic syringes containing 0.1 volume of 3.2% trisodium citrate. PRP was collected as the supernatant after centrifuging the blood at 100× *g* for 20 min at room temperature. For the generation of PPP, PRP was additionally centrifuged at 2200× *g* for 3 min. After centrifugation, the supernatant (PPP) was collected.

### 4.4. Viability Tests

PRP was incubated with different concentrations of frondoside A or vehicle (aqua dest.) for 30 min. Thereafter, 1 µL of calcein-AM (8 µM) and a saturating concentration of PE-labeled anti-CD42b antibody were added to 40 µL PRP for 30 min. The level of double-fluorescent events was assessed by means of a FACScan flow cytometer (Becton Dickinson, San Jose, CA, USA) using the CellQuest software. 

### 4.5. Assessment of pTT

PPP (100 μL) was preincubated with pTT reagent (HYPHEN BioMed, Neuville-sur-Oise, France) for 3 min at 37 °C, and the reaction was started by adding 100 µL (25 mM) CaCl_2_. The pTT was determined as the time interval between the addition of CaCl_2_ to the solution and the formation of a fibrin clot under stirring conditions. Normal and abnormal plasma served as controls. 

### 4.6. Western Blot Analysis

PRP was incubated for 30 min with different concentrations of frondoside A or vehicle (aqua dest.), and then stimulated with ADP (10 µM), PAR-1-AP (2.5 µM), or PMA (100 ng/mL) for 5 min. The cells were centrifuged for 3 min by 4 °C and lysed on ice for 10 min in lysis buffer (RIPA buffer: 50 mM Tris-HCl, pH 7.2, 0.15 M NaCl, 1.0 mM EDTA, 0.1% SDS, 1.0% Triton X-100, 1.0% sodium deoxycholate, and phosphatase inhibitor). Cell extracts were separated through a 10% SDS polyacrylamide gel and immunoblotted with specific antibodies. Protein expression was visualized via luminol-enhanced chemiluminescence (ECL; GE-Healthcare, Freiburg, Germany) and exposure of the membranes to a blue light-sensitive autoradiography film (Hyperfilm ECL, GE-Healthcare).

### 4.7. Analysis of Surface Adhesion Protein Expression 

PRP was treated with different concentrations of frondoside A or vehicle (aqua dest.) for 30 min and then stimulated with ADP (10 µM), PAR-1-AP (2.5 µM) or PMA (100 ng/mL) for 5 min. Aliquots of 10 µL PRP were then incubated with saturating concentrations of PE-labeled primary antibodies or control antibody (isotype control) for 30 min. Subsequently, the cells were fixed in 1% formalin for 10 min at 4 °C and measured using a FACScan flow cytometer (Becton Dickinson) using the CellQuest software. The levels of CD62P and GPIIb/IIIa of 100,000 cells were determined by analyzing the mean fluorescence intensity. Non-stimulated PRP served as controls.

### 4.8. Animals

For the in vivo experiments, we used male and female BALB/c mice with a body weight of 22–25 g. The animals were housed in a temperature-controlled environment under a 12 h/12 h light–dark cycle, and received standard pellet food (Altromin, Lage, Germany) and water ad libitum. All experiments were approved by the local governmental animal protection committee (Landesamt für Verbraucherschutz, Abteilung C Lebensmittel- und Veterinärwesen, Saarbrücken, Germany) and were conducted in accordance with the European legislation on protection of animals (Guideline 2010/63/EU) and the NIH guidelines for the care and use of laboratory animals (http://oacu.od.nih.gov/regs/index.htm. 8th Edition; 2011). 

### 4.9. Photochemically Induced Thrombus Formation

To analyze the effect of frondoside A on photochemically induced thrombus formation, we used the mouse dorsal skinfold chamber model [40]. For this purpose, 12 BALB/c mice were anesthetized with an intraperitoneal injection (i.p.) of 100 mg/kg ketamine and 12 mg/kg xylazine. Subsequently, dorsal skinfold chambers were implanted, as previously described [41]. To avoid alterations of the microcirculation due to anesthesia or surgical trauma, the mice were allowed to recover from the implantation procedure for 72 h. Thereafter, the animals were treated with 800 µg/kg i.p. (corresponding to a concentration of ~0.6 µM in the circulation) frondoside A (*n* = 6), vehicle (aqua dest. i.p.; *n* = 6), or 5 mg/kg i.p. clopidogrel (*n* = 6) both 15 h and 1 h before photochemically induced thrombus formation. For in vivo microscopy, mice were immobilized on a Plexiglas stage and the dorsal skinfold chamber was attached to the microscopic stage. After retrobulbary injection of 0.05 mL 5% FITC-labeled dextran 150,000 for contrast enhancement by staining of blood plasma and rhodamine 6G for in situ staining of platelets, intravital epi-illumination fluorescence microscopy was performed using a Zeiss microscope (Zeiss, Oberkochen, Germany) with a 100 W mercury lamp attached to a blue filter. The microscopic images were recorded by a charge-coupled device video camera (FK6990; Pieper, Schwerte, Germany) and transferred to a monitor (Trinitron; Sony, Tokyo, Japan) and DVD system (DVD-HR775; Samsung, Eschborn, Germany) for offline evaluation. Using a 20-fold long distance objective (Achroplan 0.50 W; Zeiss), baseline blood flow was monitored in individual postcapillary and collecting venules (diameter range: 15–25 μm; *n* = 4 per chamber). Subsequently, thrombus formation was photochemically induced by a continuously applied local exposure of the vessels to filtered light (450–490/>520 nm excitation/emission wavelength) with a 63-fold water immersion objective (Achroplan 0.95 W; Zeiss) [41,42].

Quantitative analyses of the microscopic images were performed using the offline analysis system CapImage (Zeintl, Heidelberg, Germany). Diameters and centerline RBC velocities were determined in venules prior to thrombus induction. Diameters (d) were measured in µm perpendicularly to the vessel path. Centerline RBC velocities (v, given in µm/s) were analyzed using the line shift method [43]. The kinetics of thrombus formation were assessed by measuring the time (given in s) until sustained cessation of blood flow due to complete vessel occlusion.

### 4.10. Tail Vein Bleeding Time

The tail vein bleeding time was determined as a parameter of primary hemostasis, as previously described [44]. Briefly, an incision was made over a lateral tail vein at a constant position of the tail. Subsequently, the tail was immersed in saline solution (37 °C). The time from incision to complete cessation of the blood stream was measured as bleeding time. At the end of the in vivo experiments, the animals were sacrificed by means of cervical dislocation.

### 4.11. Statistical Analysis

Data were tested for normal distribution and equal variance. Differences between two groups were analyzed by the Mann–Whitney rank sum test. Differences between multiple groups were analyzed by ANOVA (parametric data) or ANOVA on ranks (non-parametric data) followed by the Student–Newman–Keuls post hoc test (SigmaPlot 11.0; Jandel Corporation, San Rafael, CA, USA). All values are expressed as mean ± standard error (SD). Statistical significance was accepted for a value of *p* < 0.05.
